# Development and characterization of an immortalized nasopharyngeal epithelial cell line to explore airway physiology and pathology in yak (*Bos grunniens*)

**DOI:** 10.3389/fvets.2024.1432536

**Published:** 2024-07-17

**Authors:** Jiancheng Qi, Jizong Zhang, Fangyuan Huang, Yue Xie, Hongrui Guo, Liping Gou, Zhicai Zuo, Jing Fang

**Affiliations:** ^1^Key Laboratory of Animal Disease and Human Health of Sichuan Province, College of Veterinary Medicine, Sichuan Agricultural University, Chengdu, Sichuan, China; ^2^Department of Pharmacy and Pharmaceutical Sciences, Faculty of Science, National University of Singapore, Singapore, Singapore

**Keywords:** yak, nasopharyngeal epithelial cell, isolation, immortalization, cell identification, IBRV

## Abstract

Airway epithelial cells play a crucial role in investigating the physiological and pathological mechanisms of the respiratory tract in yaks, a species whose unique respiratory system has garnered extensive interest. Despite this growing interest, there currently are no available airway epithelial cell lines from yaks, underscoring the crucial need to establish a yak respiratory epithelial cell line. Therefore, our objective was to isolate a population of primary yak nasopharyngeal epithelial cells (pYNE) and transform them into immortalized yak nasopharyngeal epithelial cells (iYNE), assessing their suitability as an *in vitro* model. Employing a combined method of physical elimination and differential adhesion, we successfully isolated a population of high-purity pYNE, and developed an iYNE line through pCI-neo-hTERT plasmid transfection. Karyotype and transmission electron microscopy analyses confirmed that pYNE and iYNE share identical morphologies and structures. Gel electrophoresis and real-time PCR analyses demonstrated that pYNE and iYNE expressed similar levels of *KRT18* and *CDH1* genes (*p* ≥ 0.541). Notably, iYNE expressed a significantly high level of *TERT* gene expression (*p* < 0.001). Immunofluorescence analysis demonstrated that both cell types expressed Pan-Cytokeratin, ZO-1, and E-cadherin proteins. Furthermore, immunoblotting analysis indicated significantly higher levels of hTERT and Ki67 proteins in iYNE (*p* < 0.001), and similar levels of Cluadin-3 and Occludin proteins (*p* ≥ 0.103). Proliferation curve analysis highlighted iYNE’s serum-dependency and significantly enhanced proliferation capacities (*p* < 0.001). Additionally, pYNE and iYNE cells demonstrated comparable susceptibilities to infectious bovine rhinotracheitis virus (IBRV). These findings collectively suggest that the developed iYNE retains the evaluated physiological characteristics of pYNE, making it an appropriate *in vitro* model. This advancement will facilitate further investigation into the respiratory physiological and pathological mechanisms in yaks.

## Introduction

The yak (*Bos grunniens*), an indigenous herbivore, thrives on the Qinghai-Tibet Plateau, playing a crucial role in the lives of local communities by providing milk, meat, leather, and fuel. Beyond its economic value, the yak is central to maintaining ecosystem stability, ensuring livelihood security, fostering socio-economic development, and preserving the rich ethnic and cultural traditions in the region (1). Adapted to the plateau’s unique and harsh conditions, characterized by such as hypoxia, coldness, and long-term pasture shortage (1–3), yaks exhibit distinctive physiological traits. Notably, compared to the plains-dwelling yellow cattle, yaks possess a respiratory system with higher density of mucosal goblet cells, submucous glands, and tracheal cartilage (4, 5). Our previous study also identified significant differences in the nasopharyngeal microflora between yaks and typical cattle (6). Serving as an exemplary model for studying adaptations to high-altitude living (3), yaks offer invaluable insights into respiratory physiological regulations and the pathogenesis of pathogen infection in animals residing at high elevations (7). However, despite a few involvement of yak alveolar macrophages (8) and alveolar epithelial cells (9), the scientific community has yet to develop primary or cell lines from the yak’s respiratory system for experimental studies, underscoring the critical need for accessible and stable cell lines from this unique respiratory system.

The nasopharynx, a key segment of the upper respiratory tract, links the auditory tubes, esophagus, and trachea. It stands at the frontline of pathogens exposure and antigen sampling, serving as a critical gateway for respiratory health (10). Blanketing the palate, the nasopharyngeal mucosa hosts a diverse microbiota, encompassing both commensal and (opportunistic) pathogenic microorganisms (11, 12). The equilibrium among these microflora is predominantly maintained by the nasopharyngeal epithelium’ functionality, underscoring its significance in safeguarding cattle’s respiratory well-being (12, 13). Therefore, investigating the physiological and pathological dynamics of the yak’s nasopharyngeal epithelium holds promise for advancing effective preventive and therapeutic approaches for respiratory ailments in this species.

Airway epithelial cells play a pivotal role in research focused on understanding the regulatory mechanisms of airway mucosa barrier functions and respiratory infections in cattle (14, 15). For instance, Bai et al. (16) isolated a population of nasopharyngeal mucosal epithelial cells from bovines and confirmed its potential as an *in vitro* model for studying acute foot-and-mouth disease virus infection. In Bai’s study, which is the only available study involving nasopharyngeal epithelial cells form bovine, the isolated cells were cobblestone-shaped, grew in a single adherent layer, and expressed Pan-Cytokeratin. The process of collecting airway mucosa tissue, coupled with the isolation and purification of primary epithelial cells, particularly from the yak nasopharyngeal epithelium, presents considerable challenges and incurs significant costs (16, 17). An important consideration in working with primary cells cultured *in vitro* is their limited proliferation capacity, known as the “Hayflick Limit” (18), which constrains their utility in experimental research that necessitates multiple cell passages. For instance, research conducted by Jonathan and colleagues (19) demonstrated that primary bronchial epithelial cells could undergo only 3–4 passages *in vitro*. To overcome this obstacle, scientists have achieved success in immortalizing primary yak ruminal epithelial cells and bovine intestinal epithelial cells. This was accomplished by introducing the exogenous human telomerase reverse transcriptase (*hTERT*) gene through methods such as lentivirus-mediated gene transfer and the use of a pCI-neo-hTERT recombinant plasmid (20, 21). As a result, the overexpression of the *hTERT* gene has emerged as a dependable approach for generating immortalized cell lines.

Hence, this study is dedicated to establishing a nasopharyngeal epithelial cell line, after conducting thorough identification procedures, to serve as an *in vitro* model for exploring both the physiological functions and pathogenic interactions within the yak’s nasopharyngeal mucosa. Our study will create a reliable cell-based tool that could advance our understanding of how to prevent and treat respiratory diseases in yaks. Meanwhile, this research can provide valuable insights and methodologies for isolating, culturing, and immortalizing primary cells from various sections of the yak respiratory system.

## Materials and methods

### Nasopharyngeal mucosa tissue collection

**Figure 1:**
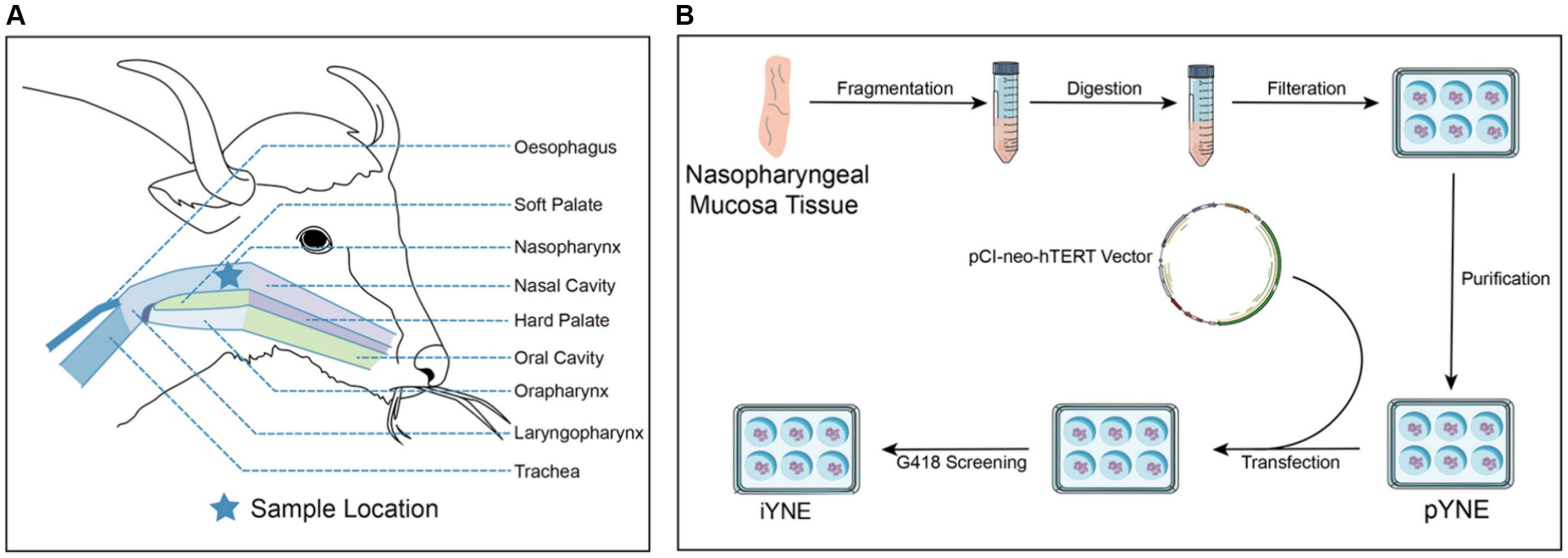
Detailed schematic of the yak’s upper respiratory tract anatomy **(A)** alongside the steps involved in developing an immortalized yak nasopharyngeal epithelial cell line **(B)**. pYNE, primary yak nasopharyngeal epithelial cells; iYNE, immortalized yak nasopharyngeal cells; G418, geneticin.

At Huirun Livestock Slaughter Co., Ltd., located in Dujiangyan City, Sichuan, China, we acquired the head of a freshly slaughtered 3-year-old male Jiulong yak. The head was bisected, and approximately 10 cm of nasopharyngeal mucosa tissue was harvested from the upper palate (Figure 1A). This sample underwent a rigorous cleaning process, being washed 6 times with ice-cold normal saline (Solarbio, Beijing, China) enhanced with 300 IU/mL Penicillin–Streptomycin (PS; Sigma-Aldrich, Saint Louis, MO, United States). Subsequently, the tissue was immersed in warm (37°C) phosphate-buffered saline (PBS; Solarbio, Beijing, China) containing 100 IU/mL PS. This preparation was swiftly transported to a biosafety cabinet within 30 min for the isolation of primary yak nasopharyngeal epithelial cells (pYNEs).

### Isolation, purification, and culture of pYNEs

The mucosa tissue was carefully segmented into 1 mm^3^ pieces and then treated with a solution of 1 mg/mL type IV collagenase (Solarbio, Beijing, China) at a volume 5 times that of the tissue at 37°C for 30 min. Afterwards, an equal volume of Dulbecco’s Modified Eagle Medium: Nutrient Mixture F12 medium (DMEM/F12; Thermo Fisher Scientific, Shanghai, China) containing 15% fetal bovine serum (FBS; Thermo Fisher Scientific, Shanghai, China) was introduced to halt the digestion process. Subsequently, the resulting cell suspension was collected and passed through a 200-mesh cell filter to remove undigested tissue.

After filtering, the cell suspension underwent centrifugation at 1,000 revolutions per minute (RPM) for 5 min at a temperature of 4°C. The collected cell sediment was then subjected to further digestion in 5 mL of 0.25% trypsin (Solarbio, Beijing, China) within an incubator enriched with 5% CO_2_ at 37°C for 2 min. To terminate the digestion, 10 mL of complete medium was added. This medium comprised 83% DMEM/F12, 15% FBS, 100 UI/mL PS, 1% Insulin-Transferrin-Selenium (ITS-G; Procell, Wuhan, China), and 20 ng/mL human epidermal growth factor (hEGF; Novoprotein, Suzhou, China). The cell suspension was then centrifuged at 1,000 RPM for 3 min at 4°C, discarding the supernatant. The resultant cell pellet was resuspended in the complete medium and seeded into 25 cm^2^ culture flasks (Nest, Wuxi, China) at a density of approximately 2 × 10^5^ cells per flask and incubated at 37°C in an incubator with 5% CO_2_ for subsequent purification process.

The cell purification process was guided by methodologies outlined in previous studies (17, 21). The condition of the cells within the flask was monitored daily. Upon reaching approximately 70% confluence, the medium was removed, and the cells were gently washed 3 times with PBS to ensure cleanliness. Subsequently, under microscopic examination, the regions containing epithelial cell clusters were identified and marked on the bottom of the culture flask. Untargeted cells (fibroblasts) in the non-target areas were then carefully eliminated using a cell scraper and further cleansed with 3 PBS rinses to eliminate fibroblasts. Afterward, fresh medium was introduced into the flask to continue cultivating the remaining epithelial cells. This procedure was repeated daily until the epithelial cells constituted over 80% of the flask’s area. In the final iteration, the remaining epithelial cells in marked regions were detached using a solution of 0.25% trypsin-0.02% EDTA (Solarbio, Beijing, China), collected via centrifugation, and then reseeded into a 6-well plate (Nest, Wuxi, China) for incubation. After a 45-min incubation period, the medium was cautiously transferred to a new 6-well plate. This transfer process was repeated 5 times to achieve a collection of purified pYNEs.

For subculturing, the cells were detached using a solution of 0.25% trypsin-0.02% EDTA upon reaching 80%–90% confluence, which typically occurred after about 3 days. This was followed by centrifugation, resuspension in fresh medium, and reseeding into new flasks.

### Plasmid transfection and selecting of immortalized nasopharyngeal epithelial cell

The purified pYNEs were evenly distributed into a 6-well plate, with each well containing approximately 2 × 10^4^ cells. Once the pYNEs reached 70%–90% confluence, they underwent transfection with 4 μg of the pCI-neo-hTERT recombinant plasmid (Supplementary Figure 1; JingMingHaoRui, Chengdu, China) and 10 μL of Lipofectamine 2000 (Thermo Fisher Scientific, Shanghai, China), maintained at 37°C for a duration of 24 h. Following this, the existing medium was replaced with a fresh complete medium containing 500 μg/mL of geneticin (G418; Beyotime Biotechnology, Shanghai, China) to select positive cell clones. These selected clones were then expanded by cultivating in a 5% CO_2_ incubator at 37°C, thus establishing an iYNE cell line.

Upon verification that this cell line was mycoplasma-free using a Mycoplasma PCR Detection Kit (C0310S, Beyond, Shanghai, China), the iYNE cell line was cryopreserved in liquid nitrogen. Currently, this cell line has exceeded over 70 passages, qualifying it as “immortal” by contemporary scientific standards (22). Figure 1B illustrates the process involved in developing the iYNE cell line.

### Karyotypic analysis

When the cells achieved 80% confluence, they were treated with 0.1 μg/mL colchicine (Solarbio, Beijing, China) and incubated at 37°C for 6 h. Subsequently, the cells were digested and collected by centrifugation at 200 g for 5 min. The resulting cell pellet was then resuspended in 75 mM KCl at room temperature and allowed to stand for 8 min. Following this, the suspension was mixed with Carnoy’s solution (methanol: glacial acetic acid, 3:1 v/v; Solarbio, Beijing, China) and maintained at 4°C before being subjected to centrifugation at 200 g. This resuspension-mixture-centrifugation procedure was repeated twice. Afterward, 100 μL of the cell suspension was dispensed onto a precooled slide and left to air-dry. The cells underwent a subsequent digestion with 0.25% trypsin-0.02% EDTA at 30°C for 10–20 min. Chromosomes were then stained with Giemsa dye (Solarbio, Beijing, China) for 10 min. The number and morphology of each chromosome in metaphase (n = 100) were thoroughly analyzed under a microscope and digitally captured.

### Transmission electron microscopy analysis

Cells forming monolayer were detached using a solution of 0.25% trypsin-0.02% EDTA. Following detachment, the detached cells were collected by centrifugation at 200 g for 15 min, washed with precooled PBS, and centrifugated at 200 g for 15 min again. Then, the resulting cell pellet underwent fixation with 2.5% glutaraldehyde (pH 7.4; Lilai Biotechnology, Chengdu, China) for 1 h. After an additional wash with precooled PBS, the cells were subjected to a second fixation using 10 g/L osmium acid for 2 h. Subsequently, the samples were dehydrated in a graded series of ethanol concentrations, embedded in epoxy resin, sectioned, and stained with uranium salts. The prepared samples were examined under a transmission electron microscope (Hitachi, Tokyo, Japan), and images were captured for further analysis.

### Gene expression analysis

Cells were seeded into 6-well plates at a density of 2 × 10^5^ cells/mL, with each well receiving 2 mL of the cell suspension. Upon reaching 80%–90% confluence, the cells were collected, and total RNA was extracted using Trizol Reagent (TransGen, Beijing, China), adhering to the manufacturer’s protocol. The RNA’s concentration and purity were assessed using a NanoDrop ND-2000 spectrophotometer (Thermo Fisher Scientific, Wilmington, NC, United States), while its integrity was verified via 1% agarose gel electrophoresis. Following this, total RNA samples were reverse transcribed to complementary DNA (cDNA) using the RevertAid First Strand cDNA Synthesis Kit (Thermo Fisher Scientific, Shanghai, China), as per the manufacturer’s instructions. Subsequently, specific genes, namely *TERT* (coding hTERT), *CDH1*(coding E-cadherin), *VIM* (coding vimentin), *KRT18* (coding CK-18), and *ACTB* (coding β-actin), were amplified from the cDNA template via polymerase chain reaction (PCR). The products of this amplification were then visualized using gel electrophoresis. To quantify the expression levels of *TERT*, *CDH1*, and *KRT18* genes relative to *ACTB* real-time PCR was employed. The analysis of real-time PCR data was conducted using the 2^ΔΔC^ method in EXCEL. Primer sequences for both PCR and real-time PCR are presented in Table 1.

**Table 1:** The sequences of primers used in the polymerase chain reaction experiments.

Gene name	Primer sequences (5′ to 3′)	Product length (bp)
*TERT*	F: AAACCTTCCTCAGCTATGCCC	219
	R: GCACACATGCGTGAAACCTG	
*CDH1*	F: ATCCAGAAACGGGTGCCATT	121
	R: GTGGCAGGTGGAGAACCATT	
*VIM*	F: GCGCTCAAAGGGACTAACGA	624
	R: GCACAAGGCGTCTTCGGTAA	
*KRT18*	F: GGAAGTGGAGGCCCGATACG	21
	R: GCGTCGCCAAGACTGAAATC	
*ACTB*	R:TCACGGAGCGTGGCTACAG	61
	F: TTGATGTCACGGACGATTTCC	

### Immunofluorescence and immunoblotting analyses

For immunofluorescence analysis, cells were seeded onto sterile coverslips in 24-well plates at a density of 5 × 10^4^ cells/mL, with 1 mL of cell suspension added to each well. The cells on the coverslips were fixed with formaldehyde (Life iLab Bio, Shanghai, China) at −20°C for 10 min. Subsequently, the formaldehyde-fixed cells were permeabilized with 0.1% Triton X-100 (Solarbio, Beijing, China) for 10 min at room temperature, blocked with 10 g/L bovine serum albumin (Solarbio, Beijing, China) for another 10 min, and incubated overnight at 4°C with the primary antibodies. The primary antibodies used in this experiment included Pan-Cytokeratin (Cat No: bs-1712R; Thermo Fisher Scientific, Shanghai, China), Zonula Occludens protein 1 (ZO)-1 (Cat No: A20532; ABclone Technology, Wuhan, China), E-cadherin (Cat No: A3044; ABclone Technology, Wuhan, China), and Ki67 (Cat No: A11390; ABclone Technology, Wuhan, China). After washing 3 times with PBS, the secondary antibody (donkey anti-rabbit IgG antibody, Cat No: ab6701; Abcam, Shanghai, China) was added and incubated at room temperature for 4 h. The cell nuclei were stained with 4′,6-diamidino-2-phenylindole (DAPI; Solarbio, Beijing, China) for 5 min at room temperature. Cells were then observed and digitally imaged using a fluorescence microscope (Olympus, Tokyo, Japan).

For immunoblotting analysis, cellular proteins were extracted from cells in each well and denatured using RIPA lysis buffer at 100°C for 10 min. An equal amount of protein was then separated using sodium dodecyl sulfate-polyacrylamide gels and transferred to a polyvinylidene fluoride (PVDF) membrane. After incubation with primary antibodies, the membranes were hybridized with a secondary antibody (donkey anti-rabbit IgG antibody, Cat No: ab6701, Abcam, Shanghai, China) and subsequently incubated with enhanced chemiluminescent reagents to visualize the protein bands. The primary antibodies used in this experiment included GAPDH (Cat No: 200306; ZEN-BIOSCIENCE, Chengdu, China), Ki67 (Cat No: 16667; Abcam, Shanghai, China), hTERT (Cat No: 32020; Abcam, Shanghai, China), Occludin (Cat No: 502601; ZEN-BIOSCIENCE, Chengdu, China), and Claudin-3 (Cat No: 14487; Abcam, Shanghai, China). The band intensity of the images was measured using the ImageJ software.

### Cell growth dynamics analysis

Cells with different treatments were seeded into 96-well plates at a concentration of 1.5 × 10^4^ cells/mL with 100 μL of cell suspension added to each well. Six replicates were established per timepoint for each treatment group, and viable cell counts were measured daily using a cell counting kit-8 (CCK-8; Nanjing Jiancheng Bioengineering Institute, Nanjing, China) following the manufacturer’s instructions. In brief, the complete medium in the wells was replaced with 100 μL of a mixture containing 90% complete medium and 10% CCK-8 reagent. The mixture was then incubated for 2 h at an incubator set at 5% CO_2_ and 37°C. After incubation, the absorbance at 450 nm for each well was measured using a microplate reader (Thermo Fisher Scientific, Waltham, MA, United States). Cell growth curves were plotted with time on the X-axis and mean optical density (OD)_450nm_ on the Y-axis.

### Infectious bovine rhinotracheitis virus susceptibility analysis

IBRV-positive viral fluid, sourced form a virulent strain of IBRV clinically isolated from the nasopharynx of a diseased yak diagnosed with respiratory infection and previously identified as IBRV by PCR, was utilized in our study. Initially, approximately 3 × 10^5^ pYNE and iYNE cells were seeded into 2 T25 flasks, respectively. Upon reaching approximately 70% confluence, the medium was discarded. Concurrently, 1 mL of the IBRV-positive viral fluid was evenly divided and introduced into each flask, where it remained for a 2-h incubation period. Following this, the viral fluid was removed, and each flask was replenished with 5 mL of complete medium to continue the culture. Observations and documentations of the morphological changes in pYNE and iYNE cells were conducted at 24 and 48 h post-infection. Additionally, the 50% tissue culture infective doses (TCID50) of IBRV for both pYNE and iYNE cells were calculated using the Reed-Muench method, as outlined in a previous study (23).

### Statistical analysis

Data are presented as the mean ± standard error (SE). Statistical analyses were performed using SPSS 26 (IBM Corporation, Armonk, NY, United States). Graphs were generated using Origin 2024 Pro (Originlab, Northampton, MA, United States), Adobe Photoshop 2022, and Adobe Illustrator 2022 (Adobe Systems Incorporated, San Jose, CA, United States), unless stated otherwise. Statistical differences were determined using the Student t-test (all data satisfy both normal distribution and variance alignment), unless specified otherwise. A *p*-value of less than 0.05 was considered statistically significant.

## Results

### Isolation and culture of pYNE and subculture of iYNE cell line

**Figure 2:**
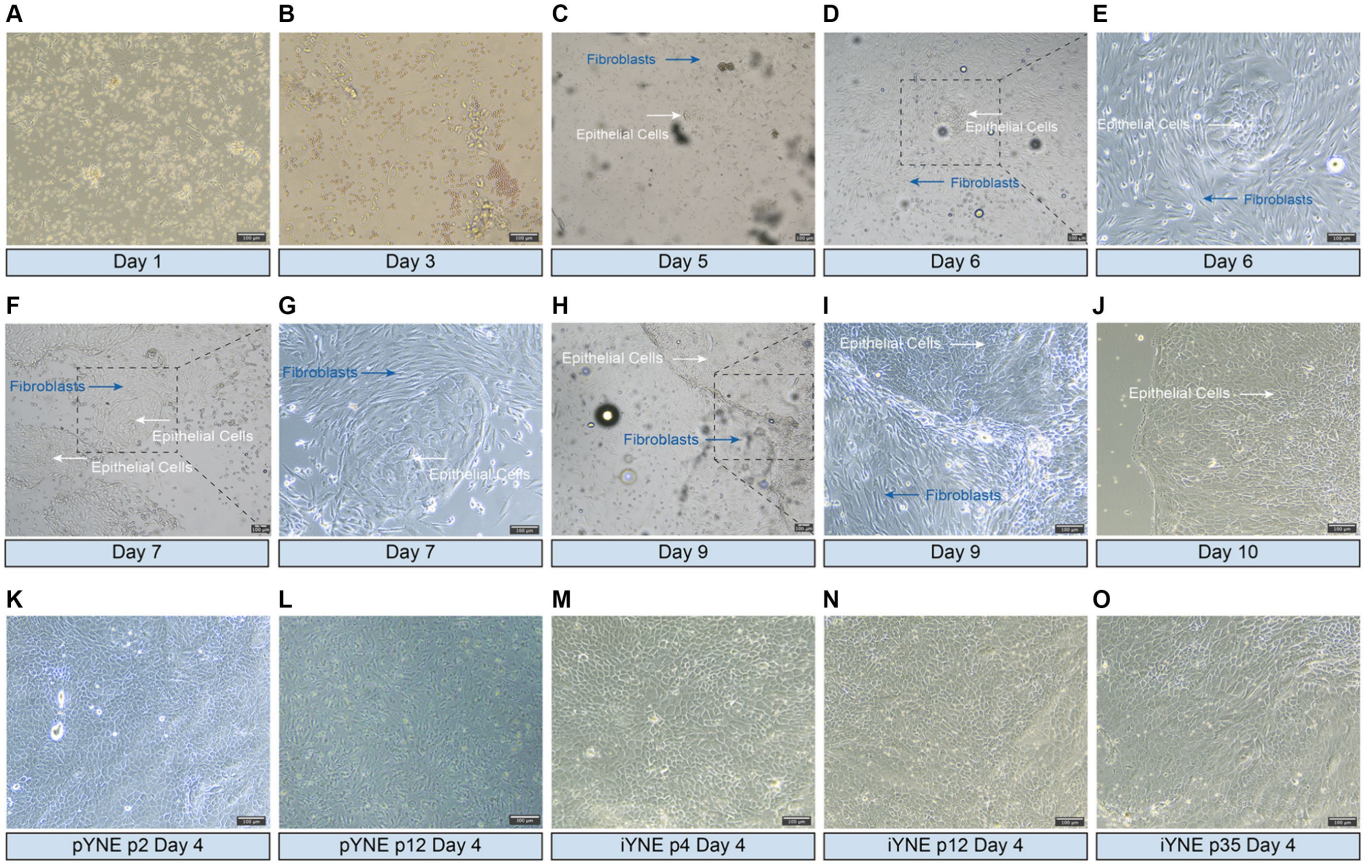
Representative images showcasing the morphologies of isolated and immortalized nasopharyngeal cells. **(A–J)** Representative images presenting the morphologies of the first passage of cells, highlighting both epithelial cells (white arrows) and fibroblasts (blue arrows) observed over days 1–10 after seeded into flask; **(K,L)** Representative images presenting the morphologies of purified epithelial cells from the 2nd **(K)** and 12th **(L)** passages of primary yak nasopharyngeal epithelial cells (pYNE) observed on the 4th day after seeded into flask; **(M–O)** Representative images presenting the morphologies of cells from the 4th **(M)**, 12th **(N)**, and 35th **(O)** passages of immortalized yak nasopharyngeal epithelial cells (iYNE) observed on the 4th day after seeded into flask.

On the 5th day after seeding into the flask, a cluster of epithelial cells was first observed, displaying a cobblestone-like appearance and forming a tightly connected, epithelioid monolayer (Figures 2A–C). By the 6th day, these cells had reached to approximately 70% confluence (Figures 2D,E). Throughout the purification process, fibroblasts were progressively eliminated, leading to a gradual increase in the number of epithelial cells (Figures 2F,I). By the 10th day, almost all fibroblasts had been removed, leaving behind a pure population of epithelial cells in the flask (Figure 2J). After undergoing 5 additional rounds of purification through the differential adhesion method, purified pYNE at the 2nd passage were obtained (Figure 2K). Following 11 passages of subculture, these cells began to exhibit obvious signs of senescence, such as altered morphology, increased intercellular gaps, and accumulation of cellular debris (Figure 2L). After transfection with pCI-neo-hTERT recombinant plasmid and selection with G418, a G418-resistant cell clone was obtained from the 4th passage of pYNE. This clone retained the same cobblestone-like appearance, tight connections, and epithelioid monolayer morphology (Figure 2L), which remained consistent through subsequent subcultures at the 12th and 35th passages (Figures 2N,O).

### Organelle morphological characterization

**Figure 3:**
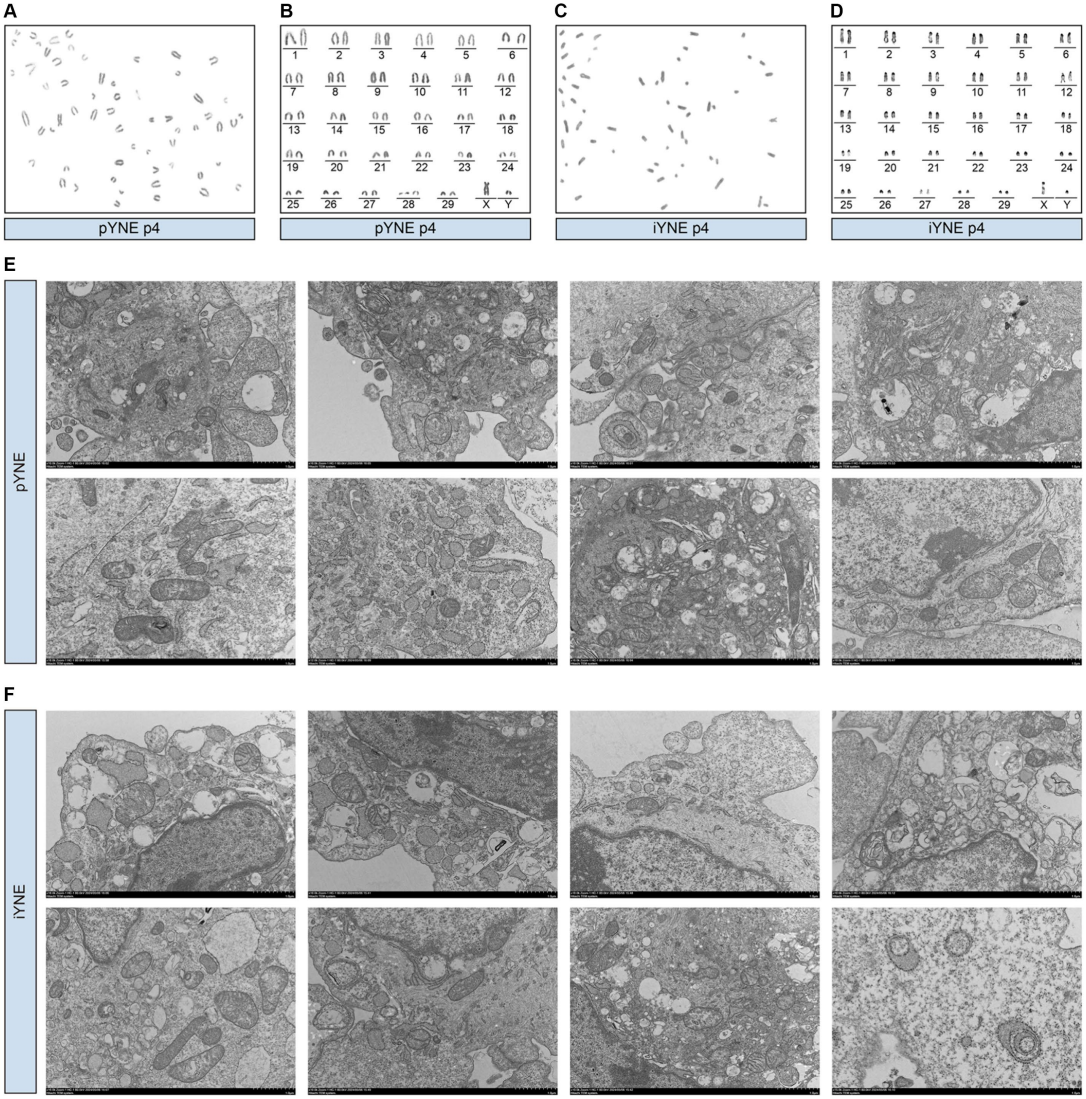
Morphological characterization of organelles in primary yak nasopharyngeal epithelial cells (pYNE) and immortalized yak nasopharyngeal epithelial cells (iYNE). **(A,B)** Images presenting the karyotyped chromosomes **(A)** and rearranged chromosomes **(B)** in the pYNE; **(C,D)** Images presenting the karyotyped chromosomes **(C)** and rearranged chromosomes **(D)** in the iYNE; **(E,F)** Images presenting the intracellular structures of pYNE **(E)** and iYNE **(F)** captured by transmission electron microscopy.

In addition to the overall morphological observation of the obtained cells, we also assessed the structure and morphology of the organelles of pYNE and iYNE by karyotype and TEM analyses. Karyotype analysis results showed that both the pYNE and iYNE contained 29 pairs of autosomes and 1 pair of XY chromosomes (Figures 3A–D). Furthermore, pYNEs and iYNEs exhibited similar sizes and shapes of mitochondria, nucleus, endoplasmic reticulum, and Golgi apparatus, and both expressed lysosomes and cytoskeleton (Figures 3E,F).

### Expression assessment of marker genes

**Figure 4:**
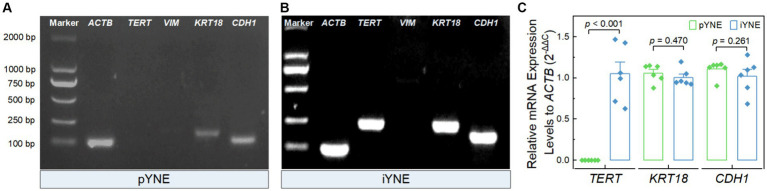
Expression files of key marker genes in pYNE and iYNE. **(A,B)** Gel images presenting the expression patterns of the marker genes *TERT*, *VIM*, *KRT18*, and *CDH1* in pYNE **(A)** and iYNE **(B)**, respectively; **(C)** Bar chart with dot presenting the relative expression levels of *TERT*, *KRT18*, and *CDH1* against *ACTB* in both pYNE and iYNE. In panel **(C)**, data are presented as mean ± standard error, and student’s t-test method was employed for statistical analysis. pYNE, primary yak nasopharyngeal epithelial cells; iYNE, immortalized yak nasopharyngeal epithelial cells.

After confirming the structure and morphology of both pYNE and iYNE, we proceeded to evaluate the expression of certain marker genes. These included the immortalization marker gene *TERT*, the fibroblast marker gene *VIM*, and epithelial cell marker genes *KRT18* and *CDH1*. The PCR results revealed that both pYNE and iYNE expressed *KRT18* and *CDH1*, but neither expressed *VIM*. The *TERT* expression was detected in iYNE but not in pYNE (Figures 4A,B). Moreover, real-time PCR analysis indicated that the level of *TERT* expression in iYNE was significantly higher than that in pYNE (*p* < 0.001), while the expression levels of *KRT18* and *CDH1* genes were similar between the two (*p* ≥ 0.541; Figure 4C).

### Expression assessment of marker proteins

**Figure 5:**
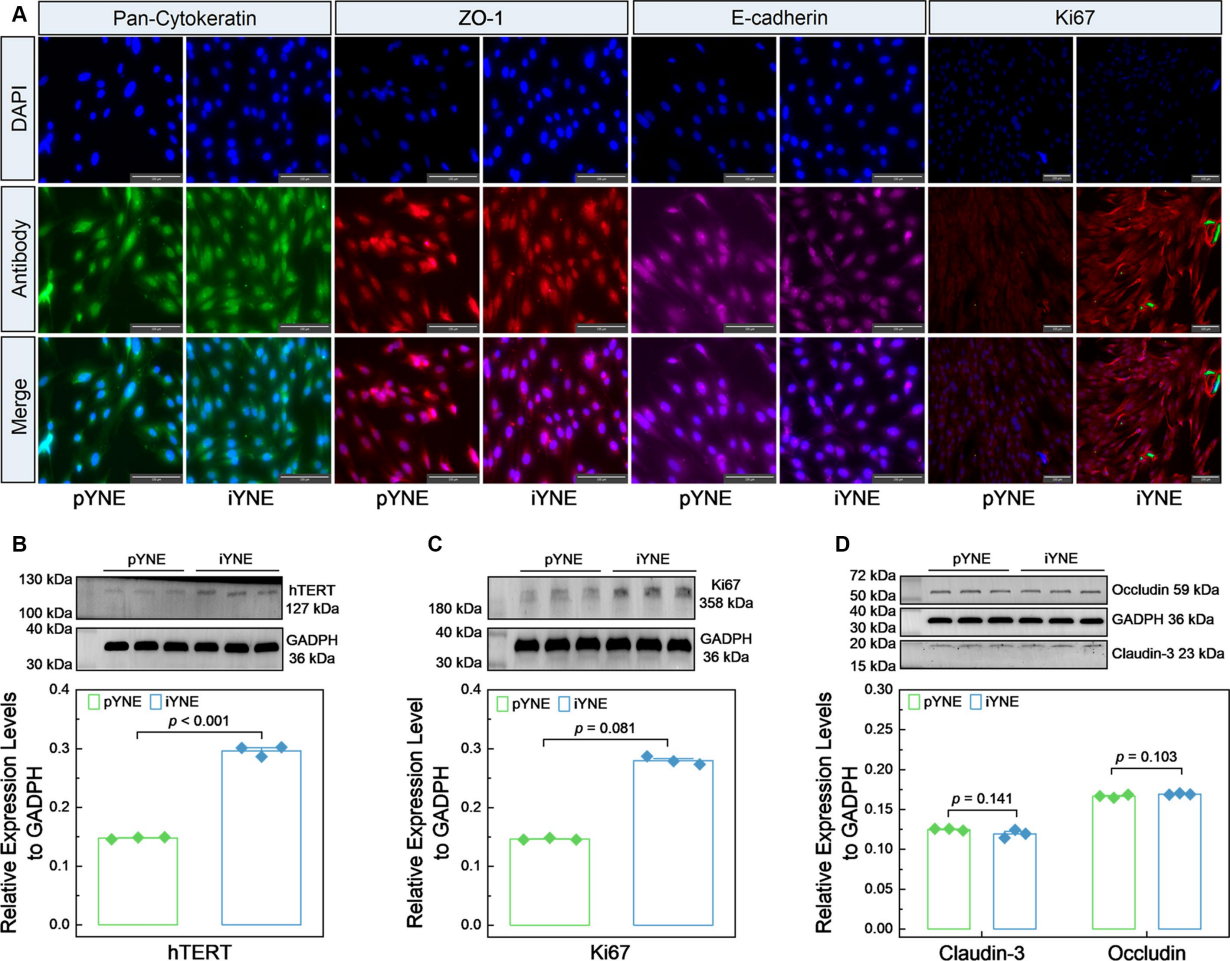
Expression files of key marker proteins in pYNE and iYNE. **(A)** Images presenting the results of immunofluorescence analysis; **(B–D)** Gel images alongside bar charts with dots presenting the immunoblotting analysis results for hTERT **(B)**, Ki67 **(C)**, Cluadin-3 and Occludin **(D)** in pYNE and iYNE. In panel **(B–D)**, data are expressed as mean ± standard error, and student’s t-test method was employed for statistical analysis. pYNE, primary yak nasopharyngeal epithelial cells; iYNE, immortalized yak nasopharyngeal epithelial cells.

We also evaluated the expression levels of various marker proteins, including epithelial cell marker proteins Pan-Cytokeratin, ZO-1, and E-cadherin, alongside the cell proliferation marker Ki67 and hTERT, in both pYNE and iYNE. As shown in Figure 5A, similar levels of Pan-Cytokeratin, ZO-1, and E-cadherin fluorescence were observed on the surface of both pYNEs and iYNEs. However, an obviously high intensity of Ki67 fluorescence was observed in iYNEs compared to pYNEs. Furthermore, immunoblotting analyses confirmed that the relative expression levels of hTERT and Ki67 were significantly higher in iYNE than in pYNE (*p* < 0.001; Figures 5B,C), whereas the relative expression levels of Claudin-3 and Occludin did not significantly differ between pYNE and iYNE (*p* ≥ 0.103; Figure 5D).

### Proliferative property assessment

**Figure 6:**
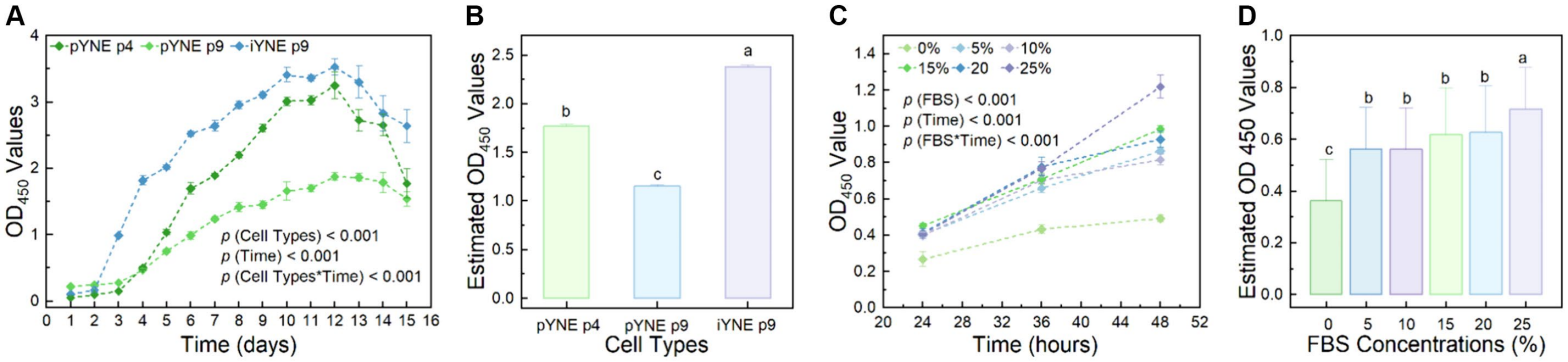
Proliferation characteristics of pYNE and iYNEs. **(A,B)** Dot-line chart and bar chart presenting the proliferation curves of pYNE and iYNE over a 15-day period **(A)** and their estimated OD values of each group **(B)**; **(C,D)** Dot-line chart and bar chart presenting the proliferation curves of iYNE treated with varying concentrations of FBS over a 48-h period **(C)** and the estimated OD values of each group **(D)**. Data are expressed as mean ± standard error, and Repeated Measures Analysis of Variance was employed to analyze the differences among groups. In panels **(B,D)**, different superscripts above the bars indicate significant differences between the corresponding groups. pYNE, primary yak nasopharyngeal epithelial cells; iYNE, immortalized yak nasopharyngeal epithelial cells; FBS, fetal bovine serum.

To further investigate the proliferation characteristics of pYNE and iYNE, we monitored their proliferation dynamics over a 15-day period post-seeding. Our observations revealed that pYNE in its 4th passage demonstrated significantly higher proliferation rates compared to its 9th passage (1.770 ± 0.022 vs. 1.153 ± 0.014; *p* < 0.001). Interestingly, the 9th passage of iYNE exhibited a markedly higher proliferation activity than the 4th passage of pYNE (2.382 ± 0.018 vs. 1.770 ± 0.022; *p* < 0.001; Figures 6A,B). Additionally, we explored the serum-dependency of iYNE’s proliferative capacities to determine its oncogenic potential. Our results revealed an enhanced proliferative activity of iYNE with increasing FBS concentrations within the 48-h period post-seeding (Figures 6C,D).

**Figure 7:**
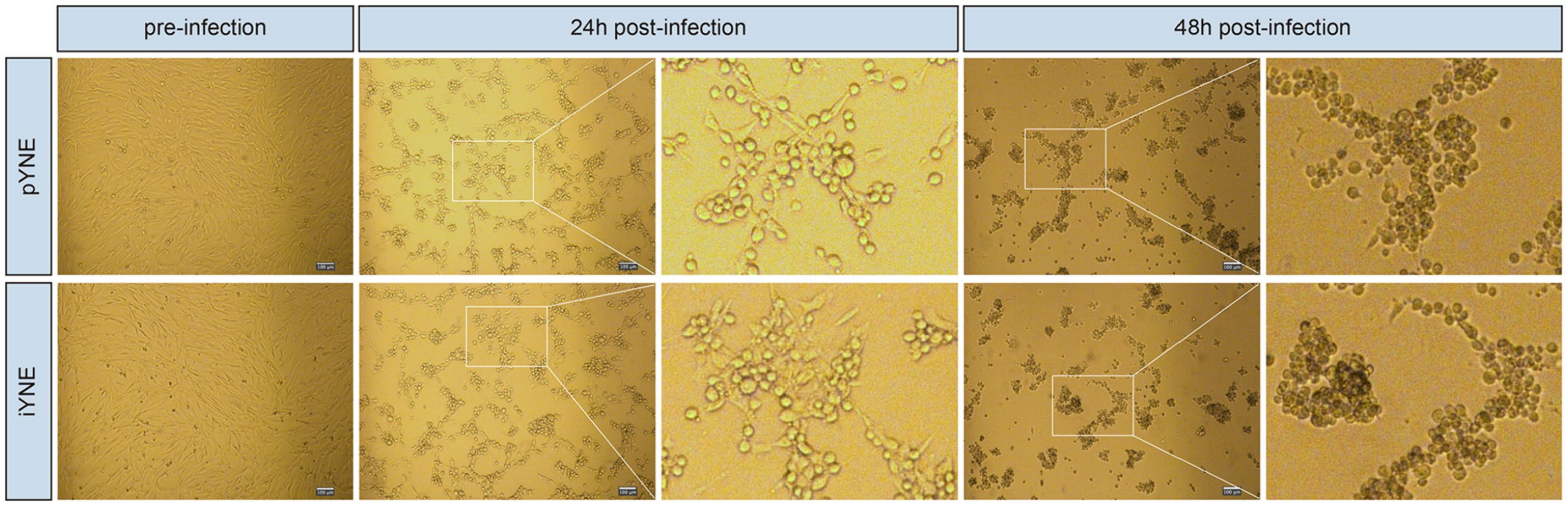
Representative images showcasing the morphologies of pYNE and iYNE pre-, 24 h post-, and 48 h post-IBRV infection. pYNE, primary yak nasopharyngeal epithelial cell; iYNE, immortalized yak nasopharyngeal epithelial cell; IBRV, infectious bovine rhinotracheitis virus.

The pYNE cells and iYNE cells were both subjected to infection by the IBRV virus. At the 24th hour post-infection, both pYNEs and iYNEs began to round up and separate from each other. Their intercellular spacing increased, and some cells started to cluster together, indicating early signs of infection. By the 48th hour, the morphologies of both cell types further deteriorated, with more cells becoming rounded and forming tight clusters. The cell clusters were more compact, with few individual cells remaining, showing pronounced signs of infection (Figure 7). Furthermore, the TCID50 for both pYNE and iYNE cells were calculated to be −5.29.

## Discussion

The yak, a species uniquely adapted to high-altitude environments, exhibits distinct morphological and physiological characteristics compared to normal cattle (5, 24, 25). Recently, the molecular mechanisms under the functions of yak alveolar cells have garnered increasing attention (8, 9). However, despite their role as the primary defense against pathogenic microorganisms in yaks (12), nasopharyngeal epithelial cells from yaks or other cattle have seldom been utilized in research except for Bai’s study (17). This oversights is likely due to the challenges associated with their isolation and limitations of their subcultural passages (17). In this study, we successfully isolated and immortalized a line of nasopharyngeal epithelial cell from a yak. We then conducted a comprehensive analysis of their physiological characteristics.

### Combined method of elimination and differential adhesion effectively purified pYNE

Bovine airway epithelial cells have become a key focus in understanding the pathogenic mechanisms behind bovine respiratory diseases (26). For instance, primary respiratory cells from both the upper and lower respiratory tracts of cattle have been isolated. These cells serve as models for investigating viral and bacterial infections (14, 27), as well as for studying the expression patterns of genes encoding antimicrobial peptides (28, 29). The isolation and purification of these primary epithelial cells were achieved through enzymatic digestion and differential adhesion methods (30). However, the use of differential adhesion for purification often necessitates multiple passages (31), and the resulting cell purity may not always be sufficient, thus significantly hindering further experiments. Given the constraints associated with the limited subcultural passages of primary airway epithelial cells, our approach involved using a cell scraper over 4 consecutive days to remove fibroblasts visible to the naked eye before proceeding with differential adhesion process. As shown in Figure 2J, by the 10th day of primary culture, the majority of cells remaining in the flask were epithelial cells. Following the differential adhesion process, no fibroblasts were observed in the flask at the 2nd passage (Figure 2K), and this purity was maintained even up to the 35th passage of iYNE (Figures 2M–O). Vimentin is a type III intermediate filament protein expressing in mesenchymal cells (32). In our study, we observed no expression of *Vimentin* gene in pYNE or iYNE, indicating that iYNE contained no fibroblasts. These findings suggest that our combined method of elimination and differential adhesion can effectively purify the cell mixture, thus facilitating the swift progression to subsequent experiments.

### iYNE exhibited significantly enhanced proliferation property

Despite the widespread use of primary airway epithelial cells in research, one of their major limitation is the restricted proliferative lifespan in culture (33). Typically, primary human airway epithelial cells slowdown in division and progressively lose the capacities to form cilia, produce mucus, or express tight junction proteins after 3–4 passages of subculture (19, 34, 35). In our study, we observed no noticeable reduction in the proliferation rate or morphological changes in the pYNE until the 9th passage. The proliferative curves indicated that the 9th passage of pYNE had a significantly lower proliferative activity compared to the 4th passage (Figures 6A,B), with noticeable morphological alterations appearing by the 12th passage (Figure 2L). These observations suggest that pYNE might maintain stability through the first 8 population doublings. This duration appears to be longer than that for airway epithelial cells from humans and may also indicated a plateau-specific respiratory function in yaks. However, comparisons with primary nasopharyngeal epithelial cells from normal cattle are needed for confirmation.

To address this limitation, numerous immortalized airway epithelial cell lines have been developed (36–38). However, immortalized airway epithelial cell lines from cattle are scarcely mentioned in the literature, with the exception of Diane Lee’s study on two lines: bovine alveolar type II and bovine 2 airway epithelia, immortalized via lentivirus-mediated gene transfection (39). Since Epstein–Barr virus, effective in immortalizing human nasopharyngeal epithelial cells (40), cannot infect bovine nasopharyngeal epithelial cells, we opted for the pCI-neo-hTERT plasmid transfection method to immortalize our pYNE (Figure 1B). Subsequent real-time PCR and immunoblotting analyses showed significantly higher relative expression levels of hTERT gene and protein (Figures 4, 5B), conforming successful transfection and expression of *TERT* gene in pYNE.

Immortalized airway epithelial cells have the potential to surpass the “Hayflick Limit” allowing for extensive subculturing. For instance, Kimberly et al. reported that their immortalized primary human airway epithelial cells, transfected with the *hTERT* gene, could be cultured for over 20 passages while remaining physiological characteristic (41). In our study, iYNE was cultured for more than 35 passages without significant morphological changes (Figures 2M–O). Despite pYNE and iYNE presented similar “S” shape growth curves, iYNE demonstrated a significantly higher proliferation activity than pYNE at an early passage (Figure 6A) and higher expression levels of Ki67 protein (Figures 5A,C), a well-known marker of cell proliferation (42). These results indicate that our pYNE has overcome the “Hayflick Limit” and exhibits enhanced proliferative properties.

### iYNE maintains the physiological characteristics and susceptibility to IBRV

Besides their proliferative properties, immortalized cells must also retain essential physiological functions from their primary counterparts to serve as effective *in vitro* models. Like the isolated nasopharyngeal mucosal epithelial from bovine in Bai’s study (17), our pYNEs and iYNEs were also cobblestone-shaped, grew in a single adherent layer (Figure 2), and expressed Pan-Cytokeratin (Figure 5A). Additionally, we also evaluated some other characteristics of respiratory epithelial cells. For example, Diane et al. established a reliable *in vitro* model that mimics the bovine lung to explore the interactions between hosts and pathogens. They verified the expression of gene and protein markers, proliferative properties, and the capacities to form films via an air-liquid interface culture system in their immortalized airway epithelial cells (39). In our research, both pYNE and iYNE exhibited a 60-chromosome diploid karyotype with no significant differences in the position and number of G-banding of autosomes or the XY chromosomes, as well as identical cellular organelles (Figure 3). CK-18, a type I keratin and crucial protein marker for identifying epithelial cells (43), along with ZO-1, occludin, and E-cadherin, key molecular markers and essential components of epithelium (30), were similarly expressed in pYNE and iYNE (Figures 4, 5). These findings suggest that iYNE successfully inherited the characteristic epithelial cell features of pYNE.

Cancerous cells often lose their serum dependence and can grow in multiple layers during unlimited passages *in vitro* (44, 45). In our study, iYNE demonstrated serum-dependence, showing low proliferative capacity under serum-free or low-serum conditions (Figures 6C,D). This observation suggests that our iYNE did not exhibit overt tumor cell characteristics after immortalization.

IBRV, a prevalent infectious disease in cattle causing persistent infection, immunosuppression, and subsequent economic losses in livestock industry (46), served as a focus for testing our iYNE’s suitability for viral infection studies. We compared the susceptibilities of pYNE and iYNE to IBRV infection and observed similar cell morphological alterations and infection processes with 48 h post-infection (Figure 7) and TCID50 values, indicating comparable IBRV susceptibilities. These findings support the use of iYNE as an *in vitro* model for IBRV infection investigations. However, the applicability of these cells in other nasopharyngeal virus infection-related studies or in understanding metabolic and physiological functions remains to be determined, necessitating further validation.

## Conclusion

In our study, we successfully isolated a high-purity population of primary nasopharyngeal epithelial cells from freshly slaughtered yaks using a combination of elimination and differential adhesion methods. Subsequently, we developed an immortalized line of yak nasopharyngeal epithelial cell to serve as a robust *in vitro* model. These immortalized cells not only retain the physiological characteristics observed in pYNE but also demonstrate significantly enhanced proliferative capacities and maintain similar susceptibility to IBRV. Our work has provided a reference for the efficient isolation of respiratory epithelial cells form cattle and resulted in the establishment of a yak nasopharyngeal epithelial cell model that is suitable for long-term culture and for studies of nasopharyngeal viral infections, thereby facilitating investigation about the respiratory physiological and pathological mechanisms in yaks.

## Data availability statement

The raw data supporting the conclusions of this article will be made available by the authors, without undue reservation.

## Ethics statement

The animal study was approved by Ethical and Welfare Committee for Animal Experiments of Sichuan Agricultural University. The study was conducted in accordance with the local legislation and institutional requirements.

## Author contributions

JQ: Conceptualization, Data curation, Formal analysis, Funding acquisition, Investigation, Methodology, Project administration, Resources, Software, Supervision, Validation, Visualization, Writing – original draft, Writing – review & editing. JZ: Conceptualization, Data curation, Investigation, Methodology, Validation, Writing – original draft, Writing – review & editing. FH: Conceptualization, Data curation, Formal analysis, Investigation, Project administration, Supervision, Writing – original draft, Writing – review & editing. YX: Investigation, Methodology, Writing – original draft, Writing – review & editing. HG: Writing – original draft, Writing – review & editing. LG: Writing – original draft, Writing – review & editing. ZZ: Conceptualization, Funding acquisition, Investigation, Methodology, Project administration, Resources, Supervision, Validation, Writing – original draft, Writing – review & editing. JF: Project administration, Resources, Validation, Writing – original draft, Writing – review & editing.

## Supplementary material

SUPPLEMENTARY FIGURE 1 Profile of the pCI-neo-hTERT plasmid used in the immortalization experiment.
