# Supplementary figures and images for: Development and characterization of an immortalized nasopharyngeal epithelial cell line to explore airway physiology and pathology in yak (Bos grunniens)
Source: Front Vet Sci. 2024 Jul 17;11:1432536. doi: 10.3389/fvets.2024.1432536 (PMC11289979; doi:10.3389/fvets.2024.1432536)

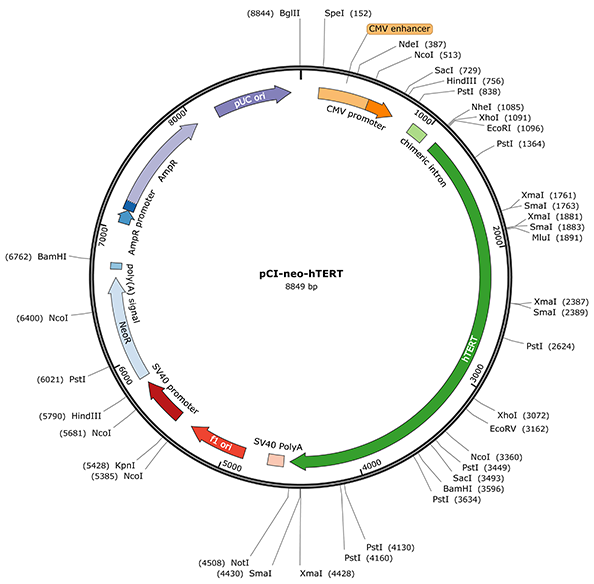

Supplement: SUPPLEMENTARY FIGURE 1 — Profile of the pCI-neo-hTERT plasmid used in the immortalization experiment. [file Image_1.tif]
